# Abortion Provision and Delays to Care in a Clinic Network in Washington State After *Dobbs*

**DOI:** 10.1001/jamanetworkopen.2024.13847

**Published:** 2024-05-29

**Authors:** Taylor Riley, Anna E. Fiastro, Lyndsey S. Benson, Anuj Khattar, Sarah Prager, Emily M. Godfrey

**Affiliations:** 1 Department of Epidemiology, University of Washington, Seattle; 2 Department of Family Medicine, University of Washington, Seattle; 3 Department of Obstetrics and Gynecology, University of Washington, Seattle; 4 Cedar River Clinics, Washington

## Abstract

**Importance:**

The Supreme Court decision *Dobbs v Jackson Women’s Health Organization (Dobbs)* overturned federal protections to abortion care and altered the reproductive health care landscape. Thus far, aggregated state-level data reveal increases in the number of abortions in states where abortion is still legal, but there is limited information on delays to care and changes in the characteristics of people accessing abortion in these states after *Dobbs.*

**Objective:**

To examine changes in abortion provision and delays to care after *Dobbs*.

**Design, Setting, and Participants:**

Retrospective cohort study of all abortions performed at an independent, high-volume reproductive health care clinic network in Washington state from January 1, 2017, to July 31, 2023. Using an interrupted time series, the study assessed changes in abortion care after *Dobbs*.

**Exposure:**

Abortion care obtained after (June 24, 2022, to July 31, 2023) vs before (January 1, 2017, to June 23, 2022) *Dobbs*.

**Main Outcome and Measure:**

Primary outcomes included weekly number of abortions and out-of-state patients and weekly average of gestational duration (days) and time to appointment (days).

**Results:**

Among the 18 379 abortions during the study period, most were procedural (13 192 abortions [72%]) and funded by public insurance (11 412 abortions [62%]). The mean (SD) age of individuals receiving abortion care was 28.5 (6.44) years. Following *Dobbs*, the number of procedural abortions per week increased by 6.35 (95% CI, 2.83-9.86), but then trended back toward pre-*Dobbs* levels. The number of out-of-state patients per week increased by 2 (95% CI, 1.1-3.6) and trends remained stable. The average gestational duration per week increased by 6.9 (95% CI, 3.6-10.2) days following *Dobbs*, primarily due to increased gestations of procedural abortions. The average gestational duration among out-of-state patients did not change following *Dobbs*, but it did increase by 6 days for in-state patients (5.9; 95% CI, 3.2-8.6 days). There were no significant changes in time to appointment.

**Conclusions and Relevance:**

These findings provide a detailed picture of changes in abortion provision and delays to care after *Dobbs* in a state bordering a total ban state. In this study, more people traveled from out of state to receive care and in-state patients sought care a week later in gestation. These findings can inform interventions and policies to improve access for all seeking abortion care.

## Key Points

**Question:**

What are the changes in abortion provision and delays to care after the Supreme Court *Dobbs v Jackson Women’s Health Organization (Dobbs)* decision in a state where abortion remained legal?

**Findings:**

This cohort study of 18 379 abortions found significant increases in the weekly number of procedural abortions, number of abortions among out-of-state individuals, and average gestational duration following the *Dobbs* decision. No significant changes were found in the time from first scheduling an appointment to receiving the abortion.

**Meaning:**

These findings suggest that more people are traveling from out of state for abortion care after *Dobbs* and that more in-state patients are obtaining abortions but are doing so approximately a week later in gestation on average.

## Teaser

This cohort study evaluates changes in abortion provision and delays to care after *Dobbs v Jackson Women’s Health Organization*.

## Introduction

The Supreme Court decision in *Dobbs v Jackson Women’s Health Organization* (*Dobbs*) in June 2022 overturned the federal right to abortion and altered the reproductive health care landscape. Following this decision, state trigger bans took immediate effect and a cascade of abortion restrictions were implemented across the country. Studies measuring the impact of *Dobbs* have reported increased out-of-state travel for abortion^1,2,3^ and increased travel time to abortion-providing facilities.^4^ In particular, the number of clinician-provided abortions has increased in states that neighbor states where abortion is restricted or banned.^1^ However, little is known about how abortion provision and delays to care may be changing in these legal states after *Dobbs*.

The longstanding inequities in abortion access, particularly at the intersections of race and class, are likely to be exacerbated after *Dobbs*.^5^ Structural factors, such as racism, sexism, and their economic consequences, underlie these inequities.^6^ Previous research documents the substantial barriers and delays to care among racially and economically marginalized individuals when abortion access was still a federally protected right.^7^ Indeed, the population health consequences of the *Dobbs* decision are expected to be dire, particularly among Black and other racially marginalized people.^8^ Thus far, the available state-level aggregated data do not provide information on any changes in the sociodemographic characteristics and delays to care among those receiving abortion care in legal states after *Dobbs*.

Washington is one of these legal states that is protective of abortion rights and borders Idaho where abortion is now completely banned. Before *Dobbs*, around 17 000 abortions were obtained in Washington per year, equivalent to a rate of 12 abortions per 1000 women aged 15 to 44 years, and around 6% of abortions were among individuals traveling from another state.^9,10^ Abortions are legal up to the point of fetal viability (as determined by clinician) or to protect the life or health of the pregnant person. Washington has had longstanding protective abortion policies such as requiring insurance plans (both Medicaid and private plans) to cover abortion and allowing abortion provision by qualified health professionals other than physicians (eg, physician assistant or advanced registered nurse practitioner).^10^ In 2023, Washington passed laws that shield clinicians who provide abortion care from investigations in other states and protect private data for people seeking abortion care.^10^ Neighboring Idaho had existing restrictive laws like mandatory waiting periods, restricted funding, and limitations on eligible clinicians before *Dobbs*, and the state’s trigger ban took effect on August 25, 2022, after the *Dobbs* decision.^10^

This study leverages a unique individual-level dataset from a large, independent abortion-providing clinic network in Washington to examine changes in abortion provision and delays to care after *Dobbs*. We conducted an interrupted time series (ITS) analysis, a robust design that controls for secular trends,^11^ to explore these temporal changes overall and stratified by race and ethnicity and by state of residence. Due to the national rapid response and increased support of resources like abortion funds^2^ that enabled more people to be able to travel for care, we hypothesized an immediate increase in the number of abortions after *Dobbs* and, consequently, increased delays to care. We also hypothesized disproportionate increases in delays among out-of-state patients due to greater travel distances and costs, and among racially marginalized individuals due to structural barriers.

## Methods

### Study Population and Data

The study population included all abortions from January 1, 2017, to July 31, 2023, performed at Cedar River Clinics, an independent, high-volume reproductive health care clinic network with 4 locations in Washington, of which 2 to 3 were open at any given time during the study period, primarily located in Western Washington. The clinic provides approximately 15% of all abortions occurring in Washington per year^12^ and provides care up to 26 weeks’ gestation. Clinic protocol requires 2 days in clinic for those at 18 weeks’ or more gestation, while procedures at 24 weeks or greater may require 2 or 3 days.

Data on patient sociodemographic characteristics and zip code of residence, type and timing of abortion, and gestational duration were extracted from electronic medical records. Data on appointment scheduling dates and calls were extracted from administrative records. These datasets were linked based on patient and encounter number identifiers (20 records, 0.1% of the study population, could not be linked to administrative data due to missing data). The University of Washington institutional review board approved the protocol for this study with exemption from informed consent because data were deidentified. This study followed the Strengthening the Reporting of Observational Studies in Epidemiology (STROBE) reporting guidelines.

### Measures

Patients self-identified their age, race and ethnicity (non-Hispanic American Indian or Alaska Native, non-Hispanic Asian, non-Hispanic Black, Hispanic or Latina, non-Hispanic Native Hawaiian or Pacific Islander, non-Hispanic White, and multiracial), primary language, reproductive history, and residential zip code when completing the patient history intake form. We conceptualized race and ethnicity as sociopolitical categories, rather than biological, that reflect historical and contemporary structural racism.^13^ Thus, race and ethnicity were assessed in this study to examine how structural racism contributes to inequities in abortion care before and after *Dobbs*. The patient’s primary payment method was categorized by the clinic’s financial staff as cash self-payment (no insurance used and includes support from abortion funds), public insurance (Medicaid), or private insurance.

The primary outcomes of interest were type of abortion (medication and procedural), gestational duration (in days), time to appointment (number of days from first contact with clinic scheduling an appointment to receiving abortion care), and patient’s state of residence (based on self-reported zip code). We conceptualized time to appointment and gestational duration as proxies for delays to care. Gestational duration was documented by clinic staff (in priority order) using ultrasound or calculated using individual-reported last menstrual period and consultation date. We performed additional data checks and cleaning for any gestational durations that were missing or over 13 weeks. Nineteen within the sample (0.1%) had documented pregnancies of unknown locations and 217 (1.2%) were missing gestational duration. These were included in the analyses of overall counts but excluded from the gestational duration analyses (described later).

### Statistical Analysis

We used standard frequency tabulation and summary statistics to report the distribution of sociodemographic and clinical characteristics of the study population in the periods before *Dobbs* (January 1, 2017, to June 23, 2022) and after *Dobbs* (June 24, 2022, to July 31, 2023). These pre-post comparisons do not account for underlying trends over time, which is particularly important given the national trends in increasing medication abortion use over the study period.^14^ Therefore, we conducted an interrupted time series (ITS) analysis to estimate changes in the outcomes of interest after the *Dobbs* decision while controlling for the overall trend in the same outcomes over time. In this study, the intervention date is the *Dobbs* Supreme Court decision on June 24, 2022. While abortion access was immediately restricted in 12 states on the day of the *Dobbs* decision, other bans or gestational limits were enacted in the following months. Therefore, for ease of interpretation, we used the *Dobbs* decision date to mark the end of federal protections for abortion (eFigure 2 in Supplement 1).

Anonymized patient-level data were aggregated into weekly counts of total number of abortions (medication and procedural), average gestational duration (days), average time to appointment (days), and total number of out-of-state patients. Segmented regression models were used to estimate both a level change (immediate change comparing before and after *Dobbs*) and a slope change for a sustained change in the outcomes of interest after *Dobbs *(eMethods in Supplement 1)*.*

We fit a segmented generalized least squares regression with Newey-West SEs to account for autocorrelation and heteroskedasticity.^11,15^ We included dummy variables (spring, summer, and fall) to control for seasonality of abortions.^16^ We assessed autocorrelation through visual inspection of complete and partial autocorrelation function plots and calculation of the Durbin-Watson statistic.^11,17,18^ Following best practices,^19^ we plotted the observed and expected temporal trends in the outcomes of interest, which were derived from the segmented regression models. To assess any differential changes associated with *Dobbs* on racialized groups and states of residence, we conducted exploratory analyses stratifying by race and ethnicity and by state of residence (out of Washington vs in Washington). For the race-stratified analysis, we combined Asian and Native Hawaiian or Pacific Islander and only included racialized groups that accounted for greater than 10% of the population for adequate sample sizes. Analyses were conducted in R Version 4.3.0 (R Project for Statistical Computing). Statistical tests were 2 sided and used *P* < .05 to indicate statistical significance.

## Results

A total of 18 379 abortions occurred during the study period, of which 3378 occurred after *Dobbs* (June 24, 2022, to July 31, 2023). The mean (SD) age of individuals receiving abortion care was 28.5 (6.44) years. Most were procedural abortions (13 192 abortions [72%]) compared with medication. A total of 2457 abortions (13%) were among Asian people, 4184 (23%) were among Black people, 2583 (14%) were among Hispanic/Latina people, and 5718 (31%) were among White people (Table 1; eFigure 1 in Supplement 1). Over half of individuals receiving abortion care had 1 or more children (11 253 individuals [61%]) and used public insurance (11 412 individuals [62%]). Most self-reported English as their primary language (16 980 individuals [92%]).

**Table 1.** Characteristics of People Receiving Abortion Care at Clinic Network in Washington State, Before *Dobbs v Jackson Women’s Health Organization (Dobbs)* (January 1, 2017, to June 23, 2022) and After *Dobbs* (June 24, 2022, to July 31, 2023)

Characteristics	Individuals, No. (%)		
	Before *Dobbs* (n = 15 001)	After *Dobbs* (n = 3378)	Total (N = 18 379)
Age, mean (SD), y	28.5 (6.41)	28.8 (6.58)	28.5 (6.44)
Race and ethnicity			
American Indian or Alaskan Native	379 (2.5)	67 (2.0)	446 (2.4)
Asian	2023 (13.5)	434 (12.8)	2457 (13.4)
Black	3460 (23.1)	724 (21.4)	4184 (22.8)
Hispanic/Latina	2011 (13.4)	572 (16.9)	2583 (14.1)
Native Hawaiian or Pacific Islander	314 (2.1)	60 (1.8)	374 (2.0)
White	4731 (31.5)	987 (29.2)	5718 (31.1)
Multiracial or other	671 (4.5)	340 (10.1)	1011 (5.5)
Declined to provide or missing	1412 (9.4)	194 (5.7)	1606 (8.7)
Self-reported primary language			
English	13 867 (92.4)	3113 (92.2)	16 980 (92.4)
Spanish	425 (2.8)	134 (4.0)	559 (3.0)
Other	709 (4.7)	131 (3.9)	840 (4.6)
Out of state	559 (3.7)	196 (5.8)	755 (4.1)
No. of children			
0	5471 (36.5)	1318 (39.0)	6789 (36.9)
1	3757 (25.0)	802 (23.7)	4559 (24.8)
≥2	5492 (36.6)	1202 (35.6)	6694 (36.4)
Missing	281 (1.9)	56 (1.7)	337 (1.8)
Primary payer			
Self-pay	2721 (18.1)	703 (20.8)	3424 (18.6)
Public insurance	9425 (62.8)	1987 (58.8)	11 412 (62.1)
Private insurance	2855 (19.0)	688 (20.4)	3543 (19.3)
Type of abortion			
Medication abortion	3790 (25.3)	1397 (41.4)	5187 (28.2)
Procedural abortion	11 211 (74.7)	1981 (58.6)	13 192 (71.8)
Fetal indication^a^	521 (3.5)	206 (6.1)	727 (4.0)
Estimated gestational duration, mean (SD), wks and d	68.3 (36.8)	68.0 (36.4)	68.2 (36.7)
≤5wk 6d	2959 (19.7)	693 (20.5)	3652 (19.9)
6wk 0d-6wk 6d	3516 (23.4)	729 (21.6)	4245 (23.1)
7wk 0d-7wk 6d	1501 (10.0)	360 (10.7)	1861 (10.1)
8wk 0d-8wk 6d	1149 (7.7)	295 (8.7)	1444 (7.9)
9wk 0d-9wk 6d	857 (5.7)	196 (5.8)	1053 (5.7)
10wk 0d-13wk 6d	1895 (12.6)	465 (13.8)	2360 (12.8)
14wk 0d-17wk 6d	1308 (8.7)	294 (8.7)	1602 (8.7)
18wk 0d-21wk 6d	953 (6.4)	159 (4.7)	1112 (6.1)
22wk 0d-23wk 6d	301 (2.0)	65 (1.9)	366 (2.0)
≥24wk 0d	356 (2.4)	92 (2.7)	448 (2.4)
Missing	206 (1.4)	30 (0.9)	236 (1.3)
Time to appointment, mean (SD), d	6.09 (7.19)	6.27 (7.62)	6.12 (7.28)

^a^ Includes fetal anomalies, genetic abnormalities, previable preterm premature rupture of membranes, and pregnancy loss.

**Figure 1.**
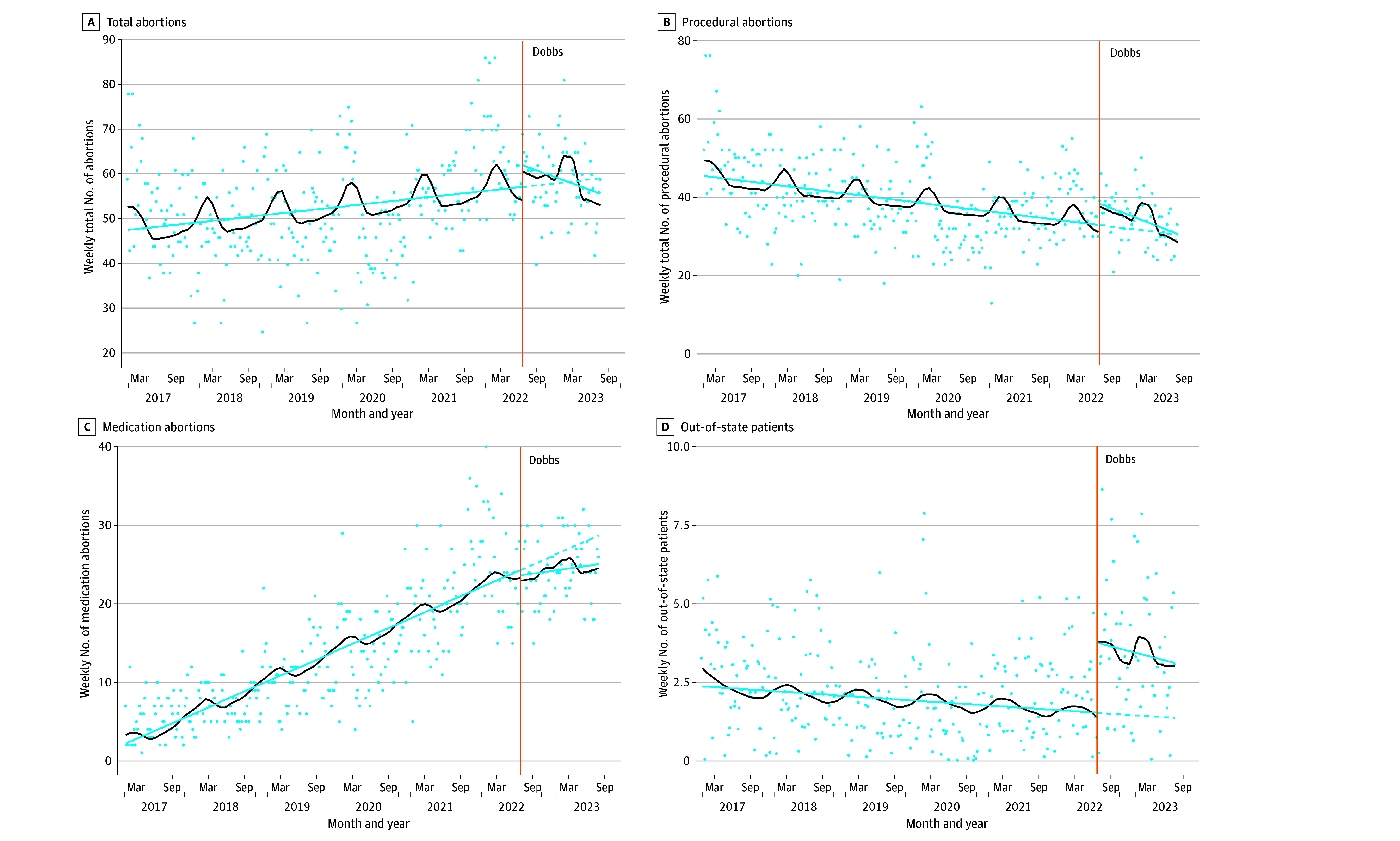
Changes in Weekly Number of Abortions and Out-of-State Patients Before and After the *Dobbs v Jackson Women’s Health Organization* Decision, January 1, 2017, to July 31, 2023 Vertical orange line indicates the Supreme Court *Dobbs v Jackson Women’s Health Organization* decision (June 24, 2022). The blue line indicates deseasonalized trends, the black line indicates estimated seasonality, the dotted line indicates the counterfactual, and the dots indicate the outcome (eg, weekly number of abortions).

**Figure 2.**
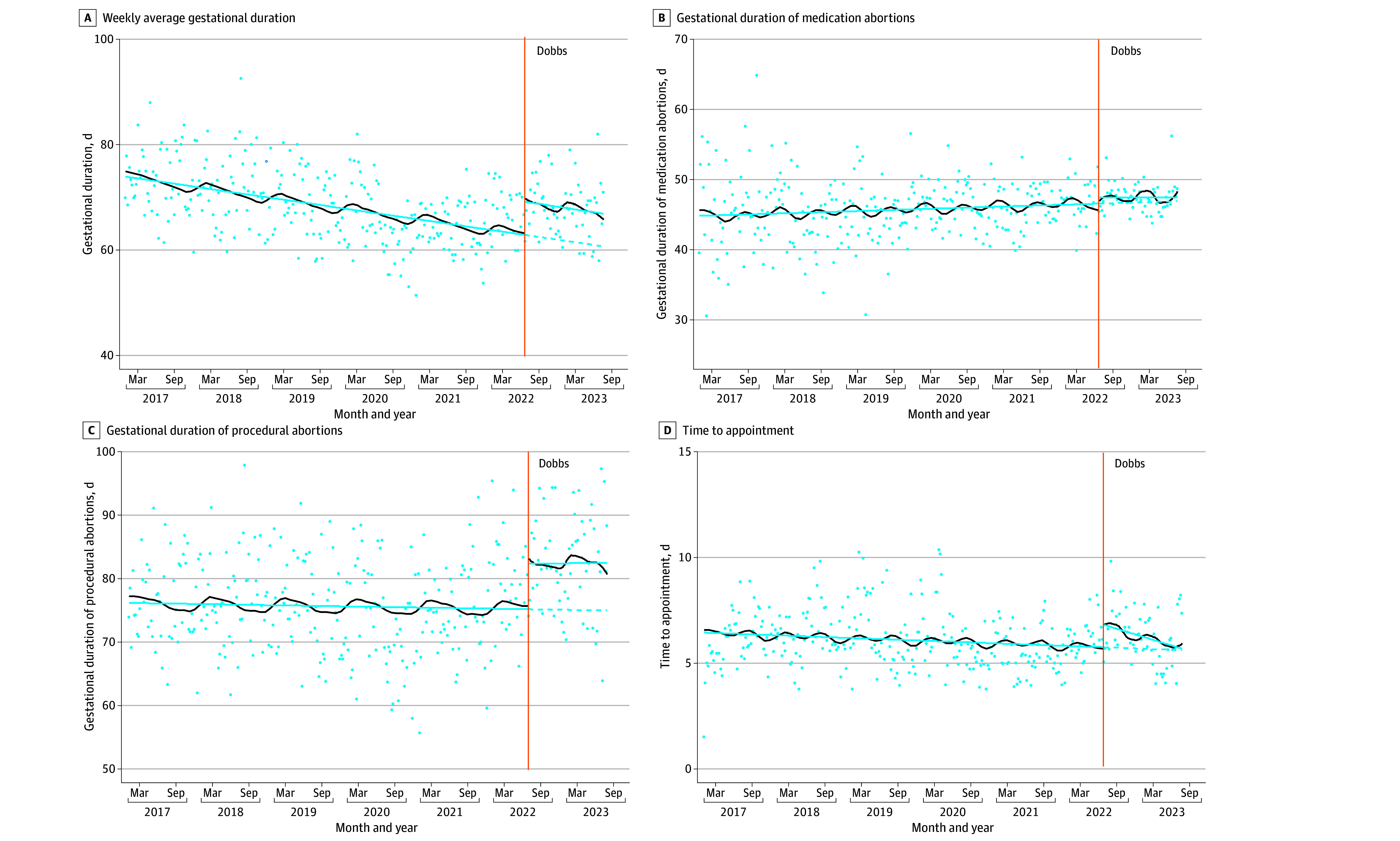
Changes in Delays to Care Before and After the *Dobbs v Jackson Women’s Health Organization (Dobbs)* Decision, January 1, 2017, to July 31, 2023 Vertical orange line indicates the Supreme Court *Dobbs* decision (June 24, 2022). A, Trend line includes all abortions (medication and procedural), which is therefore impacted by the increasing trends in medication abortion (which occur at earlier gestations) pre-*Dobbs* (see Figure 1C). B And C, gestational duration among just medication abortions (2B) and procedural abortions (2C) which shows changes in gestational duration while holding constant the type of abortion. The blue line indicates deseasonalized trends, the black line indicates estimated seasonality, the dotted line indicates the counterfactual, and the dots indicate the outcome (eg, weekly number of abortions).

ITS models showed the *Dobbs* decision was associated with increases in the weekly number of procedural abortions, average gestational duration of procedural abortions, and number of patients from out of state (Table 2, Figure 1, and Figure 2). The total weekly number of procedural abortions increased by 6.35 (95% CI, 2.83 to 9.86) following *Dobbs*. The weekly trend in number of procedural abortions decreased slightly after *Dobbs* (−0.12; 95% CI, −0.19 to −0.05) toward the level before *Dobbs*. There were no changes in the weekly number of medication abortions. The overall weekly average gestational duration increased by 6.9 days (95% CI, 3.6 to 10.2 days) following the *Dobbs* decision. The model-based average gestational duration before *Dobbs* was around 9 weeks and 4 days (67 days; 95% CI, 66 to 68 days) and after *Dobbs* was around 10 weeks and 4 days (74 days; 95% CI, 71 to 77 days). This overall increase was due to increases in the average gestational duration of procedural abortions (8.1 days; 95% CI, 3.6 to 12.6 days) and not medication abortions. The post-*Dobbs* weekly trend in average gestational duration was stable. There were no significant changes in the time to appointment following *Dobbs*. The weekly number of out-of-state patients increased by 2 (95% CI, 1.1 to 3.6) following *Dobbs*.

**Table 2.** Level and Trend Changes From Interrupted Time Series Models Before and After the *Dobbs v Jackson Women’s Health Organization (Dobbs)* Decision, January 1, 2017, to July 31, 2023

	Level change after *Dobbs*		Weekly trend after *Dobbs*	
	β (95% CI)	*P* value	β (95% CI)	*P* value
No. of abortions				
Total	5.67 (−0.07 to 11.42)	.05	−0.17 (−0.31 to −0.03)	.02
Medication abortions	−0.67 (−3.67 to 2.33)	.66	−0.05 (−0.13 to 0.03)	.21
Procedural abortions	6.35 (2.83 to 9.86)	<.001	−0.12 (−0.19 to −0.05)	.001
Delays to care, d				
Average gestational duration (total)	6.92 (3.61 to 10.23)	<.001	−0.02 (−0.1 to 0.06)	.58
Average gestational duration for medication abortion	0.54 (−0.98 to 2.05)	.49	0 (−0.04 to 0.05)	.93
Average gestational duration for procedural abortion	8.11 (3.58 to 12.64)	<.001	−0.02 (−0.12 to 0.07)	.64
Time to appointment	1.03 (−0.08 to 2.13)	.07	−0.02 (−0.06 to 0.02)	.40
No. of out of state patients	2.33 (1.1 to 3.56)	<.001	−0.01 (−0.04 to 0.02)	.43

In stratified ITS analyses, the largest increases in the average gestational duration following *Dobbs* were among Latina (8.1 days; 95% CI, 0.7 to 15.5 days), White (7.9 days; 95% CI, 3.0 to 13.0 days), and Black (4.7 days; 95% CI, 1.0 to 8.4 days) individuals (Table 3). There was an increase in average gestational duration among Asian individuals following the *Dobbs* decision but it was not significant (5.9 days; 95% CI, −0.9 to 12.6 days). While there were no significant changes in time to appointment among the entire population, stratified analyses revealed an increased time to appointment following *Dobbs* among Black (1.6 days; 95% CI, 0.3 to 2.9 days) and White (1.5 days; 95% CI, 0.3 to 2.6 days) people obtaining care.

**Table 3.** Level and Trend Changes After *Dobbs v Jackson Women’s Health Organization (Dobbs)* in Delays to Care From Stratified Interrupted Time Series Models, by Patient’s Self-Reported Race and Ethnicity and State of Residence, January 1, 2017, to July 31, 2023

Delays to care	Level change after *Dobbs*		Weekly trend after *Dobbs*	
	β (95% CI)	*P* value	β (95% CI)	*P* value
Average gestational duration, d				
Race and ethnicity				
Asian	5.89 (−0.85 to 12.63)	.08	0 (−0.15 to 0.15)	.99
Black	4.7 (1.02 to 8.37)	.01	−0.02 (−0.11 to 0.07)	.65
Hispanic/Latina	8.06 (0.67 to 15.46)	.03	0.04 (−0.16 to 0.23)	.72
White	7.94 (2.96 to 12.93)	.002	0.02 (−0.14 to 0.17)	.84
Residence				
Out of state	5.06 (−6.13 to 16.25)	.38	0.04 (−0.33 to 0.4)	.83
In state	5.92 (3.21 to 8.63)	<.001	−0.03 (−0.11 to 0.05)	.40
Time to appointment, d				
Race and ethnicity				
Asian	0.79 (−0.43 to 2.01)	.21	−0.02 (−0.05 to 0.02)	.26
Black	1.6 (0.28 to 2.91)	.02	−0.02 (−0.05 to 0.02)	.38
Hispanic/Latina	0.71 (−1.22 to 2.64)	.47	−0.02 (−0.09 to 0.04)	.44
White	1.45 (0.34 to 2.55)	.01	−0.02 (−0.06 to 0.02)	.35
Residence				
Out of state	2.71 (−0.6 to 6.03)	.11	−0.02 (−0.11 to 0.07)	.67
In state	0.96 (−0.12 to 2.04)	.08	−0.02 (−0.06 to 0.02)	.36

Around 4% of abortions (559 abortions) were among people living outside of Washington before *Dobbs* and increased to 6% (196 abortions) after *Dobbs*. A higher proportion of out-of-state patients compared with in-state were Indigenous and White (and less so Asian, Black, and Hispanic), obtained procedural compared with medication abortions, self-paid for their abortion, sought care due to a fetal indication (eg, genetic abnormalities), and had a higher gestational duration compared with in-state patients (eTable 1 in Supplement 1). The proportion of out-of-state patients who identified as Indigenous decreased substantially after *Dobbs* (from 120 [22%] to 15 [8%]). Conversely, the proportion of Black (from 43 [8%] to 42 [21%]) and Latina (from 45 [8%] to 32 [16%]) patients from out of state increased. Out-of-state patients receiving abortion care at less than 14 weeks increased by 54% and paying out of pocket increased by 67% after *Dobbs*, and there were minimal changes among in-state patients across time periods. The average gestational duration among out-of-state patients increased but was not statistically significant (5.1 days; 95% CI, −6.1 to 16.3 days) following the *Dobbs* decision, but it did significantly increase by 6 days for in-state patients (5.9 days; 95% CI, 3.2 to 8.6 days) (Table 3). We found no changes in time to appointment for either in-state or out-of-state patients.

## Discussion

Our study finds evidence of increases in procedural abortions and gestational duration among people receiving abortion services at a large abortion clinic network in Washington. Following the *Dobbs* decision, there were around 6 more procedural abortions per week on average, which were generally 8 days later in gestation, and 2 more out-of-state patients compared with the period before *Dobbs*. This aligns with other studies showing increases in the number of facility-based abortions in Washington,^1,3^ increases in individuals traveling out of state for care,^2^ and increased gestational duration after *Dobbs*.^20^ While most published studies have reported aggregated numbers, this study leverages a unique individual-level clinical and administrative dataset with a rigorous study design to present a more detailed picture of the changes in abortion provision and delays in a large clinic network. These findings also affirm the emerging evidence that the post-*Dobbs* increase in abortions in legal states is not just due to out-of-state patients but that more people living within legal states are obtaining abortions, potentially because a previously unmet need for abortion is now being met with increased resources for and attention on abortion after *Dobbs* that may impact people’s abortion decision-making (eTable 2 in Supplement 1).^1^

Our findings also provide new information about delays to care after *Dobbs*. While there were no changes in the time from first scheduling an appointment to receiving the abortion, we did find that people, primarily those living in Washington, obtained abortions almost a week later in gestation after *Dobbs*. Together, these findings suggest that people are seeking care later due to factors that are external to the clinic. There are numerous factors that could contribute to seeking care later in pregnancy, including delays in suspecting pregnancy, difficulty in deciding to terminate, primigravity, finding an abortion clinician, sorting out insurance coverage, and making financial and logistical arrangements.^21,22,23,24,25^ It is also possible that individuals first sought care at other clinics that had limited availability or longer wait times, resulting in obtaining care at this clinic network later in gestation. The *Dobbs* decision could have exacerbated these barriers, as well as added more confusion and uncertainty about the legality of abortion and where to seek care, leading to further delays.^26^ While the average increase from around 9 to 10 weeks could push people near or beyond eligibility for medication abortion, studies have found that any delay increases the risk of complications^27^ and can have negative impacts on psychological well-being.^28^ Thus, this potential early trend is concerning given the economic and health implications of later abortion care.^29,30^

We also generated new insights into the sociodemographic shifts in abortion access after *Dobbs*. The most substantial changes were among out-of-state patients with proportional increases in Black and Hispanic patients, decreases in Indigenous patients, and increases in self-payment, which could be due to increased abortion fund support as documented in other studies.^2^ Conversely, a consistent proportion of out-of-state patients sought care for fetal anomalies or abnormal pregnancies both before and after *Dobbs*, suggesting this clinic network has long been an important resource for those seeking care for this reason, which typically occurs at later gestations when abortion is restricted in other states. Regarding delays, we found increases across racialized groups but differing magnitude and statistical significance. While 1 previous study^31^ documented potential delayed recognition of pregnancy due to increased menstrual irregularities among Latina women, which could contribute to seeking care at later gestations, we were unable to explore the mechanisms of these differential delays. Importantly, these findings by racialized groups reflect structural inequities, rather than individual behavior or biology, and future research should address these underlying mechanisms.^6^ We urge cautious interpretation of these exploratory findings and encourage future research to address the sociopolitical factors that influence inequitable access and experiences with abortion care.

As clinicians and policymakers in Washington and elsewhere adapt to the new legal landscape and aim to provide supportive and prompt access to abortion care for all, these findings point to important policy and practice recommendations. First, this early trend of an approximate 1 week increase in gestational duration after *Dobbs* is worrisome given the economic and health implications of delayed care and later abortions.^24,27,28^ Strengthening existing primary care and telehealth accessibility, financial support, and referral systems within the state could address these delays by expanding accessibility to earlier abortion care. Increased public education and awareness about where and how to seek abortion care in Washington can also help reduce delays. Second, because the majority of Washington counties have no abortion-providing facility (59%), which accounts for 10% of the state’s female population,^32^ expanding the number of abortion-providing facilities in underserved areas of the state could also ameliorate delays. Lastly, while these findings are specific to a clinic network in Washington, researchers in other protective states might find this ITS analytic approach useful because it is an improvement over pre-post study designs that mask underlying trends and can help develop policy solutions that respond to context-specific changes attributable to the *Dobbs* decision.

### Limitations

This study has important limitations. This clinic network may not be generalizable to other clinical settings and the data do not include self-managed abortions; thus, these findings do not provide the full picture of abortion access in Washington. The time to appointment was calculated starting from each individual’s first call to the clinic and we do not have any information on previous care-seeking elsewhere. Additionally, using gestational duration as a proxy for delays to care is limited in capturing all aspects of delayed abortion care (eg, does not include people who were delayed and never received care). Zip codes were self-reported during appointment scheduling and confirmed at intake, but it is possible out-of-state individuals used a Washington zip code, perhaps to avoid detection or to obtain public insurance. If this is the case, the number of out-of-state patients would be underestimated. We do not have data on individual income or class status, which would better highlight economic disparities in abortion access and delays. Additionally, missingness on self-reported race and ethnicity was around 9% which could bias the stratified racialized group analyses. While ITS studies are only vulnerable to time-varying confounders and there were likely no competing interventions at the same time as the *Dobbs* decision, there were no available data or an appropriate control to minimize this potential confounding and thus there is the possibility of residual unmeasured confounding. Additionally, this study only examined trends during the first 13 months after the *Dobbs* decision, but abortion trends and the distribution of gestational duration could have changed in subsequent months given the ongoing changes to the national abortion legal and health care landscape.

## Conclusions

This study provides novel evidence of the association between *Dobbs* and delays to care and abortion provision in Washington, which borders a total-ban state (Idaho). The *Dobbs* decision was associated with significant increases in the number of procedural abortions and out-of-state patients, as well as an average week increase in gestational duration, among individuals receiving care at a large clinic network. These findings provide a detailed picture of changes in abortion care that can inform state policies and practices to improve access for all seeking abortion care.

## Notes

Supplement 1. **eTable 1. **Characteristics of Out-of-State and In-State Patients, Before and After *Dobbs*, January 1, 2017, to July 31, 2023 **eFigure 1. **Total Number of Abortions, Stratified by Self-Reported Race and Ethnicity of Patient, January 1, 2017, to July 31, 2023 **eTable 2. **Level and Trend Changes From Interrupted Time Series Models Before and After *Dobbs* Decision, In-State Washington Patients Only, January 1, 2017, to July 31, 2023 **eFigure 2. **Weekly Total Number of Abortions With Key Dates Highlighted, January 1, 2017, to July 31, 2023 **eMethods. **Analysis Details and R Script

Supplement 2.
Data Sharing Statement

## References

[1] Society of Family Planning. #WeCount Report April 2022 to June 2023. 2023. Accessed October 30, 2023. https://societyfp.org/wp-content/uploads/2023/10/WeCountReport_10.16.23.pdf

[2] Keefe-Oates B, Fulcher I, Fortin J, . Use of abortion services in massachusetts after the Dobbs decision among in-state vs out-of-state residents. JAMA Netw Open. 2023;6(9):e2332400. doi:10.1001/jamanetworkopen.2023.32400 37672274 PMC10483311

[3] Guttmacher Institute. Monthly abortion provision study. 2023. Accessed October 19, 2023. https://www.guttmacher.org/monthly-abortion-provision-study

[4] Rader B, Upadhyay UD, Sehgal NKR, Reis BY, Brownstein JS, Hswen Y. Estimated travel time and spatial access to abortion facilities in the US before and after the Dobbs v Jackson Women’s Health Decision. JAMA. 2022;328(20):2041-2047. doi:10.1001/jama.2022.20424 36318194 PMC9627517

[5] Kozhimannil KB, Hassan A, Hardeman RR. Abortion access as a racial justice issue. N Engl J Med. 2022;387(17):1537-1539. doi:10.1056/NEJMp2209737 36069823

[6] Dehlendorf C, Harris LH, Weitz TA. Disparities in abortion rates: a public health approach. Am J Public Health. 2013;103(10):1772-1779. doi:10.2105/AJPH.2013.301339 23948010 PMC3780732

[7] Dehlendorf C, Weitz T. Access to abortion services: a neglected health disparity. J Health Care Poor Underserved. 2011;22(2):415-421. doi:10.1353/hpu.2011.0064 21551921

[8] Stevenson AJ. The pregnancy-related mortality impact of a total abortion ban in the United States: a research note on increased deaths due to remaining pregnant. Demography. 2021;58(6):2019-2028. doi:10.1215/00703370-9585908 34693444 PMC10577877

[9] Washington State Department of Health. Abortion. Accessed October 5, 2023. https://doh.wa.gov/you-and-your-family/sexual-and-reproductive-health/abortion

[10] Guttmacher Institute. Interactive map: US abortion policies and access after Roe. Accessed October 5, 2023. https://states.guttmacher.org/policies/

[11] Bernal JL, Cummins S, Gasparrini A. Interrupted time series regression for the evaluation of public health interventions: a tutorial. Int J Epidemiol. 2017;46(1):348-355. doi:10.1093/ije/dyw098 27283160 PMC5407170

[12] Washington State Department of Health. Pregnancy and abortion dashboard. Accessed October 23, 2023. https://doh.wa.gov/data-statistical-reports/washington-tracking-network-wtn/pregnancy-and-abortion/county-dashboard

[13] Lett E, Asabor E, Beltrán S, Cannon AM, Arah OA. Conceptualizing, contextualizing, and operationalizing race in quantitative health sciences research. Ann Fam Med. 2022;20(2):157-163. doi:10.1370/afm.2792 35045967 PMC8959750

[14] Jones R, Nash E, Cross L, Philbin J, Kirstein M. Medication abortion now accounts for more than half of all US abortions. Guttmacher Institute; 2022. Accessed May 8, 2024. https://www.guttmacher.org/article/2022/02/medication-abortion-now-accounts-more-half-all-us-abortions

[15] Newey WK, West KD. A simple, positive semi-definite, heteroskedasticity and autocorrelation consistent covariance matrix. Econometrica. 1987;55(3):703-708. doi:10.2307/1913610

[16] Franklin TE, Theisen G, Salyer CV, Pinkston C, Gunaratnam B. The seasonality of abortion in Kentucky. Contraception. 2017;95(2):181-185. doi:10.1016/j.contraception.2016.08.019 27593333

[17] Wagner AK, Soumerai SB, Zhang F, Ross-Degnan D. Segmented regression analysis of interrupted time series studies in medication use research. J Clin Pharm Ther. 2002;27(4):299-309. doi:10.1046/j.1365-2710.2002.00430.x 12174032

[18] Gebski V, Ellingson K, Edwards J, Jernigan J, Kleinbaum D. Modelling interrupted time series to evaluate prevention and control of infection in healthcare. Epidemiol Infect. 2012;140(12):2131-2141. doi:10.1017/S0950268812000179 22335933 PMC9152341

[19] Turner SL, Karahalios A, Forbes AB, . Creating effective interrupted time series graphs: review and recommendations. Res Synth Methods. 2021;12(1):106-117. doi:10.1002/jrsm.1435 32657532 PMC7818488

[20] Trevino J, Paul R, King E, Reeves J, Madden T. P035 - change in clinic volume and gestational age at time of abortion in southern Illinois Before And After Dobbs v Jackson Women’s Health Organization. Contraception. 2023;127:110202. doi:10.1016/j.contraception.2023.110202

[21] Foster DG, Jackson RA, Cosby K, Weitz TA, Darney PD, Drey EA. Predictors of delay in each step leading to an abortion. Contraception. 2008;77(4):289-293. doi:10.1016/j.contraception.2007.10.010 18342653

[22] Jung C, Fiastro A, Cornell A, Steward R, Rible R, Gipson JD. Patient perspectives on barriers in obtaining timely abortion care in Los Angeles, California. Contraception. 2023;117:50-54. doi:10.1016/j.contraception.2022.08.003 36055362

[23] Foster DG, Gould H, Biggs MA. Timing of pregnancy discovery among women seeking abortion. Contraception. 2021;104(6):642-647. doi:10.1016/j.contraception.2021.07.110 34363842

[24] Upadhyay UD, Weitz TA, Jones RK, Barar RE, Foster DG. Denial of abortion because of provider gestational age limits in the United States. Am J Public Health. 2014;104(9):1687-1694. doi:10.2105/AJPH.2013.301378 23948000 PMC4151926

[25] Kiley JW, Yee LM, Niemi CM, Feinglass JM, Simon MA. Delays in request for pregnancy termination: comparison of patients in the first and second trimesters. Contraception. 2010;81(5):446-451. doi:10.1016/j.contraception.2009.12.021 20399953

[26] Aiken ARA, Starling JE, Scott JG, Gomperts R. Requests for self-managed medication abortion provided using online telemedicine in 30 US states before and after the Dobbs v Jackson Women’s Health Organization decision. JAMA. 2022;328(17):1768-1770. doi:10.1001/jama.2022.18865 36318139 PMC9627414

[27] Cates W Jr, Schulz KF, Grimes DA, Tyler CW Jr. 1. The effect of delay and method choice on the risk of abortion morbidity. Fam Plann Perspect. 1977;9(6):266-268, 273. doi:10.2307/2134347 923756

[28] Wasser O, Ralph L, Kaller S, Antonia Biggs M. Experiences of delay-causing obstacles and mental health at the time of abortion seeking. Contracept X. Published online March 6, 2024. doi:10.1016/j.conx.2024.100105 PMC10966177 38544923

[29] Upadhyay UD, Desai S, Zlidar V, . Incidence of emergency department visits and complications after abortion. Obstet Gynecol. 2015;125(1):175-183. doi:10.1097/AOG.0000000000000603 25560122

[30] Roberts SCM, Gould H, Kimport K, Weitz TA, Foster DG. Out-of-pocket costs and insurance coverage for abortion in the United States. Womens Health Issues. 2014;24(2):e211-e218. doi:10.1016/j.whi.2014.01.003 24630423

[31] Nobles J, Cannon L, Wilcox AJ. Menstrual irregularity as a biological limit to early pregnancy awareness. Proc Natl Acad Sci U S A. 2022;119(1):e2113762118. doi:10.1073/pnas.2113762118 34969843 PMC8740731

[32] Jones R, Witwer E, Jerman J. Abortion Incidence and Service Availability in the United States, 2017. Guttmacher Institute; 2019. doi:10.1363/2019.30760

