# Supplementary material for: Abortion Provision and Delays to Care in a Clinic Network in Washington State After Dobbs
Source: JAMA Netw Open. 2024 May 29;7(5):e2413847. doi: 10.1001/jamanetworkopen.2024.13847 (PMC11137636; doi:10.1001/jamanetworkopen.2024.13847)
Supplement: Supplement 1. — eTable 1. Characteristics of Out-of-State and In-State Patients, Before and After Dobbs, January 1, 2017, to July 31, 2023 eFigure 1. Total Number of Abortions, Stratified by Self-Reported Race and Ethnicity of Patient, January 1, 2017, to July 31, 2023 eTable 2. Level and Trend Changes From Interrupted Time Series Models Before and After Dobbs Decision, In-State Washington Patients Only, January 1, 2017, to July 31, 2023 eFigure 2. Weekly Total Number of Abortions With Key Dates Highlighted, January 1, 2017, to July 31, 2023 eMethods. Analysis Details and R Script [file jamanetwopen-e2413847-s001.pdf]

Supplemental Online Content

Riley T, Fiastro AE, Benson LS, Khattar A, Prager S, Godfrey EM. Abortion provision and delays to care in a clinic network in Washington State After Dobbs. *JAMA Netw Open*. 2024;7(5):e2413847. doi:10.1001/jamanetworkopen.2024.13847

eTable 1. Characteristics of Out-of-State and In-State Patients, Before and After Dobbs, January 1, 2017, to July 31, 2023

eFigure 1. Total Number of Abortions, Stratified by Self-Reported Race and Ethnicity of Patient, January 1, 2017, to July 31, 2023

eTable 2. Level and Trend Changes From Interrupted Time Series Models Before and After Dobbs Decision, In-State Washington Patients Only, January 1, 2017, to July 31, 2023

eFigure 2. Weekly Total Number of Abortions With Key Dates Highlighted, January 1, 2017, to July 31, 2023

eMethods. Analysis Details and R Script

This supplemental material has been provided by the authors to give readers additional information about their work.

© 2024 Riley T et al. *JAMA Network Open.*

eTable 1. Characteristics of Out-of-State and In-State Patients, Before and After *Dobbs*, January 1, 2017, to July 31, 2023

Characteristics	Out of state patients		In state (Washington) patients	
	Pre-Dobbs N = 559 No. (%)	Post-Dobbs N = 196 No. (%)	Pre-Dobbs N = 14442 No. (%)	Post-Dobbs N = 3182 No. (%)
Race and ethnicity				
American Indian/Alaskan Native	120 (21.5)	15 (7.7)	259 (1.8)	52 (1.6)
Asian	29 (5.2)	14 (7.1)	1994 (13.8)	420 (13.2)
Black	43 (7.7)	42 (21.4)	3417 (23.7)	682 (21.4)
Hispanic	45 (8.1)	32 (16.3)	1966 (13.6)	540 (17.0)
Native Hawaiian or Pacific Islander	9 (1.6)	1 (0.5)	305 (2.1)	59 (1.9)
White	221 (39.5)	70 (35.7)	4510 (31.2)	917 (28.8)
Multiracial/Other	19 (3.4)	13 (6.6)	652 (4.5)	327 (10.3)
Declined to Provide/Missing	73 (13.1)	9 (4.6)	1339 (9.3)	185 (5.8)
Type of abortion				
Medication abortion	44 (7.9)	26 (13.3)	3746 (25.9)	1371 (43.1)
Procedural abortion	515 (92.1)	170 (86.7)	10696 (74.1)	1811 (56.9)
Fetal indication*	59 (10.6)	20 (10.2)	462 (3.2)	186 (5.8)
Primary payer				
Self-pay	231 (41.3)	135 (68.9)	2490 (17.2)	568 (17.9)
Public insurance	252 (45.1)	39 (19.9)	9173 (63.5)	1948 (61.2)
Private insurance	76 (13.6)	22 (11.2)	2779 (19.2)	666 (20.9)
Estimated gestational duration (days), Mean (SD)	115 (45.6)	102 (47.7)	66.4 (35.2)	65.9 (34.5)
Up to 5w6d	34 (6.1)	12 (6.1)	2925 (20.3)	681 (21.4)
6w0d-6w6d	45 (8.1)	21 (10.7)	3471 (24.0)	708 (22.3)
7w0d-7w6d	20 (3.6)	15 (7.7)	1481 (10.3)	345 (10.8)
8w0d-8w6d	17 (3.0)	17 (8.7)	1132 (7.8)	278 (8.7)
9w0d-9w6d	18 (3.2)	7 (3.6)	839 (5.8)	189 (5.9)
10w0d-13w6d	56 (10.0)	31 (15.8)	1839 (12.7)	434 (13.6)
14w0d-17w6d	85 (15.2)	16 (8.2)	1223 (8.5)	278 (8.7)
18w-21w6d	154 (27.5)	44 (22.4)	799 (5.5)	115 (3.6)
22w0d-23w6d	62 (11.1)	13 (6.6)	239 (1.7)	52 (1.6)
24w0d and above	63 (11.3)	20 (10.2)	293 (2.0)	72 (2.3)
Missing	5 (0.9)	0 (0)	201 (1.4)	30 (0.9)
Time to appointment (days), Mean (SD)	7.08 (6.28)	7.68 (7.17)	6.05 (7.23)	6.19 (7.64)

*Note: Pre-Dobbs (January 1, 2017-June 23, 2022) and Post-Dobbs (June 24, 2022- July 31, 2023). w=week, d=day. *Includes fetal anomalies, genetic abnormalities, previable preterm premature rupture of membranes, pregnancy loss.*

© 2024 Riley T et al. *JAMA Network Open.*

eFigure 1. Total Number of Abortions, Stratified by Self-Reported Race and Ethnicity of Patient, January 1, 2017, to July 31, 2023

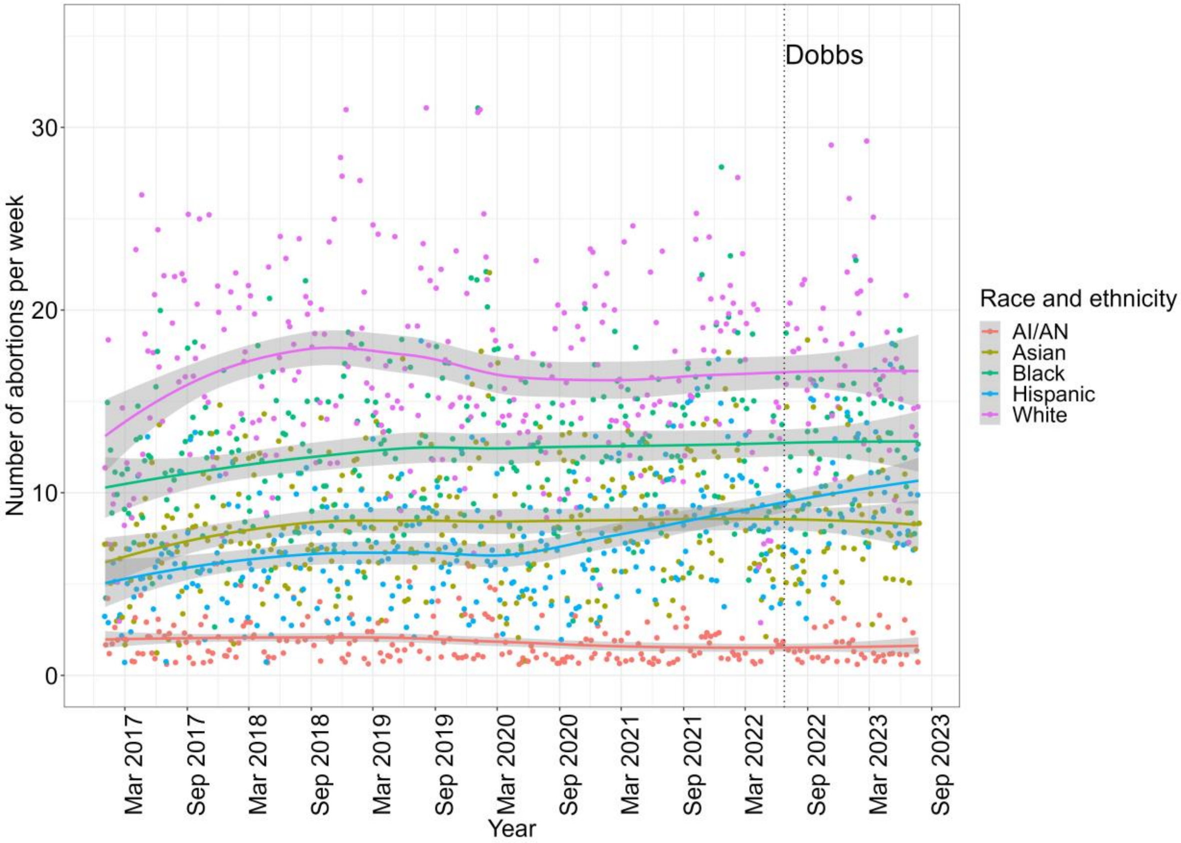

AI/AN = American Indian and Alaska Native

eTable 2. Level and Trend Changes From Interrupted Time Series Models Before and After Dobbs Decision, In-State Washington Patients Only, January 1, 2017, to July 31, 2023

	Level change after *Dobbs**		Weekly trend after *Dobbs**	
	Beta (95% CI)	p value	Beta (95% CI)	p value
**Number of abortions**				
Total	3.34 (-2.79, 9.47)	0.286	**-0.16 (-0.29, -0.02)**	**0.031**
Medication abortions	-0.69 (-3.75, 2.37)	0.658	-0.05 (-0.13, -0.03)	0.211
Procedural abortions	**4.04 (0.32, 7.75)**	**0.034**	**-0.11 (-0.19, -0.02)**	**0.014**

© 2024 Riley T et al. *JAMA Network Open.*

eFigure 2. Weekly Total Number of Abortions With Key Dates Highlighted, January 1, 2017, to July 31, 2023

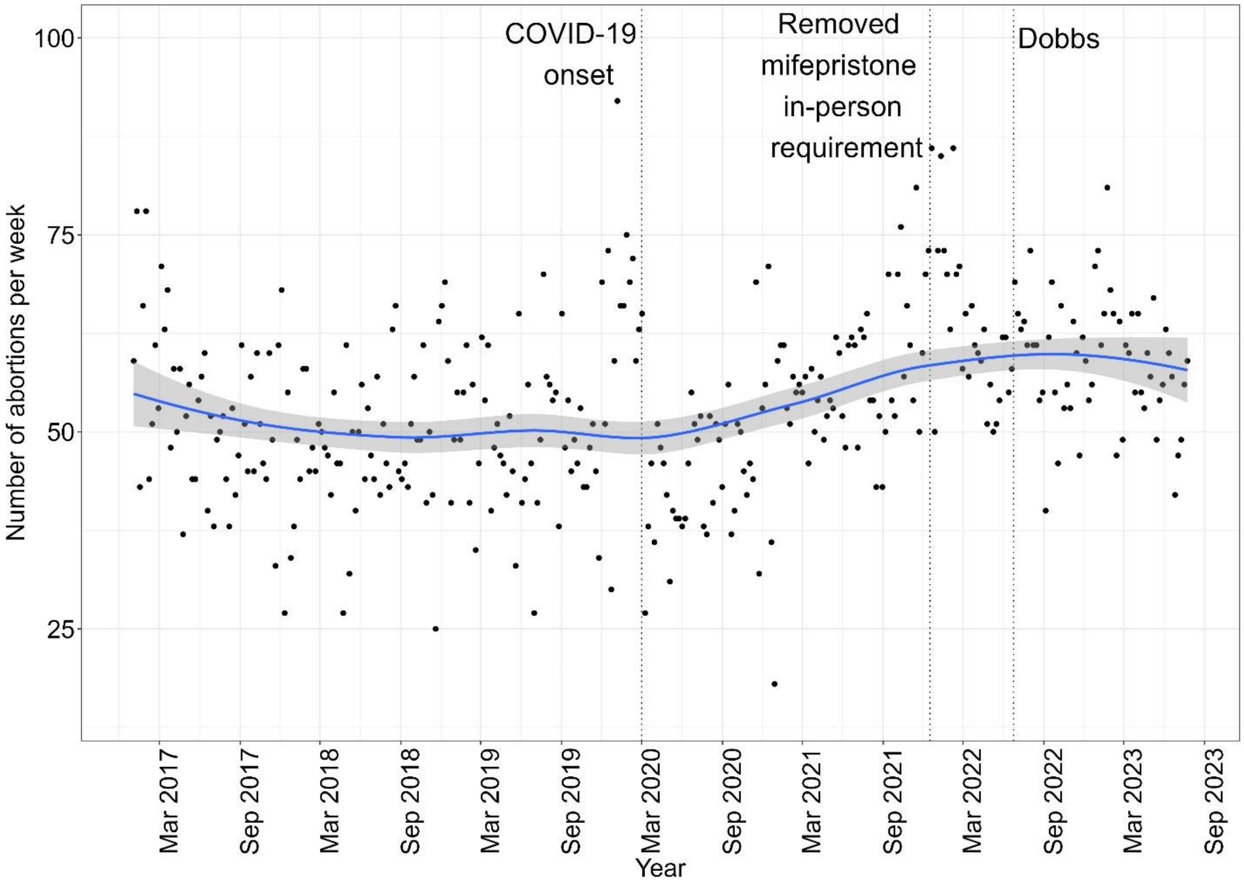

© 2024 Riley T et al. *JAMA Network Open.*

eResults. Analysis Details and R Script

1. Below is the formula used for the interrupted time series analysis:

$$Y_t = B_0 + B_1 T + B_2 X_t + B_3 T X_t$$

where T: the time elapsed since start of the study in weeks, X_t: dummy variable indicating pre-*Dobbs* period (coded 0) or post-*Dobbs* period (coded 1), and Y_t: outcome at time *t.*

2. Below is the R script for the ITS analysis with the example outcome of total number of weekly abortions.

```
##################################
# Total number of abortions
##################################
##########
# Primary Model
##########
m1 <- lm(tot ~ time_weeks + dobbs_time + time_after + winter + spring + summer,
         data = wk.yr)

# Autocorrelation
dwtest(m1)

res1 <- residuals(m1,type="deviance")
plot(wk.yr$time_weeks, res1,pch=19,cex=0.7,col=grey(0.6),
     main="Residuals over time",ylab="Deviance residuals",xlab="Date")
abline(h=0,lty=2,lwd=2)
acf(res1)
pacf(res1)
```

© 2024 Riley T et al. *JAMA Network Open.*

```
# Save model

m1tab <- coeftest(m1,vcov=NeweyWest(m1,verbose=T))


# Predict

pred.m1 <- predict(m1, wk.yr)

wk.yr <- cbind(wk.yr, pred.m1)


##########

# Counterfactual

##########


# Create a new dataset where Treatment and Time Since Treatment are equal to 0 as the intervention
did not occur.

counter.m1 <- wk.yr %>%

  mutate(

    dobbs_time = 0,

    time_after = 0,

  ) %>%

  select(tot, yr_wk, time_weeks, dobbs_time, time_after, winter, spring, summer)


# Predict the counterfactuals

cpred.m1 <- predict(m1, counter.m1)

counter.m1 <- cbind(counter.m1, cpred.m1)


##########

# Deseasonalized

##########

# Deseasonalized

m1.des <- lm(tot ~ time_weeks + dobbs_time + time_after,
```

© 2024 Riley T et al. *JAMA Network Open.*

```
            data = wk.yr)

pred.m1.des <- predict(m1.des, wk.yr)
wk.yr <- cbind(wk.yr, pred.m1.des)

# Deasonalized counterfactual
cpred.m1.des <- predict(m1.des, counter.m1)
counter.m1 <- cbind(counter.m1, cpred.m1.des)

##########
# Plot
##########
tot <- wk.yr %>%
  ggplot(aes(x = as.Date(yr_wk), y = tot)) +
  theme(
    panel.background = element_rect(fill = 'white', colour = 'black'),
    text = element_text(size = 16),
    axis.text.x = element_text(angle = 90, vjust = 0.5, hjust=1)
  ) +
  xlab("") +
  ylab("Weekly total number of abortions") +
  geom_point(size=1, alpha = .7, color = "#2b8cbe") +
  # DOBBS vertical line
  ylim(20, 90)+
  geom_vline(xintercept = as.numeric(as.Date("2022-06-24")), linetype = "dashed", color = "red")+
  annotate(geom="text", as.Date("2022-10-05"), y=90, label="Dobbs",size = 5)+
  scale_x_date(date_labels = "%b %Y", date_breaks = "6 month") +
  # Seasonalised trend - ACTUAL
  geom_smooth(data = filter(wk.yr, as.Date(yr_wk)< "2022-06-24"),
```

© 2024 Riley T et al. *JAMA Network Open.*

```
aes(x = as.Date(yr_wk), y = pred.m1), color = "grey", linewidth = 1, method = "loess", se = F, span = .1) +
  geom_smooth(data = filter(wk.yr, as.Date(yr_wk)> "2022-06-24"),
            aes(x = as.Date(yr_wk), y = pred.m1), color = "grey", linewidth = 1, method = "loess", se = F, span = .3) +
  # Deasonalised trend - ACTUAL
  geom_line(data = filter(wk.yr, as.Date(yr_wk)< "2022-06-24"),
          aes(x = as.Date(yr_wk), y = pred.m1.des), color = "#2b8cbe", linewidth = 1) +
  geom_line(data = filter(wk.yr, as.Date(yr_wk)> "2022-06-24"),
          aes(x = as.Date(yr_wk), y = pred.m1.des), color = "#2b8cbe", linewidth = 1) +
  # Counterfactual if Dobbs didnt happen
  geom_line(data = filter(counter.m1, as.Date(yr_wk)> "2022-06-24"),
          aes(x = as.Date(yr_wk), y = cpred.m1.des), lty = 2, color = "#2b8cbe", linewidth = 1)
```

© 2024 Riley T et al. *JAMA Network Open.*
