# Supplementary material for: Abortion Provision and Delays to Care in a Clinic Network in Washington State After Dobbs
Source: JAMA Netw Open. 2024 May 29;7(5):e2413847. doi: 10.1001/jamanetworkopen.2024.13847 (PMC11137636; doi:10.1001/jamanetworkopen.2024.13847)
Supplement: Supplement 2. — Data Sharing Statement [file jamanetwopen-e2413847-s002.pdf]

Data Sharing Statement

Riley. Abortion Provision and Delays to Care in a Clinic Network in Washington State After Dobbs. *JAMA Netw Open*. Published May 29, 2024.
doi:10.1001/jamanetworkopen.2024.13847

Data
Data available: No

Additional Information
Explanation for why data not available: Data will not be shared due to patient privacy concerns given the stigmatized nature of abortion care.
